# Mouse connective tissue mast cell proteases tryptase and carboxypeptidase A3 play protective roles in itch induced by endothelin-1

**DOI:** 10.1186/s12974-020-01795-4

**Published:** 2020-04-22

**Authors:** Elín I. Magnúsdóttir, Mirjana Grujic, Jessica Bergman, Gunnar Pejler, Malin C. Lagerström

**Affiliations:** 1 Department of Neuroscience, Uppsala University, Husargatan 3, Box 593, 751 24 Uppsala, Sweden; 2 Department of Medical Biochemistry and Microbiology, Uppsala University, Uppsala, Sweden; 3 Department of Anatomy, Physiology and Biochemistry, Swedish University of Agricultural Sciences, Uppsala, Sweden

**Keywords:** Itch, Chymase, Carboxypeptidase A3, Tryptase, Mice

## Abstract

**Background:**

Itch is an unpleasant sensation that can be debilitating, especially if it is chronic and of non-histaminergic origin, as treatment options are limited. Endothelin-1 (ET-1) is a potent endogenous vasoconstrictor that also has the ability to induce a burning, non-histaminergic pruritus when exogenously administered, by activating the endothelin A receptor (ET_A_R) on primary afferents. ET-1 is released endogenously by several cell-types found in the skin, including macrophages and keratinocytes. Mast cells express ET_A_Rs and can thereby be degranulated by ET-1, and mast cell proteases chymase and carboxypeptidase A3 (CPA3) are known to either generate or degrade ET-1, respectively, suggesting a role for mast cell proteases in the regulation of ET-1-induced itch. The mouse mast cell proteases (mMCPs) mMCP4 (chymase), mMCP6 (tryptase), and CPA3 are found in connective tissue type mast cells and are the closest functional homologs to human mast cell proteases, but little is known about their role in endothelin-induced itch.

**Methods:**

In this study, we evaluated the effects of mast cell protease deficiency on scratching behavior induced by ET-1. To investigate this, mMCP knock-out and transgenic mice were injected intradermally with ET-1 and their scratching behavior was recorded and analyzed.

**Results:**

CPA3-deficient mice and mice lacking all three proteases demonstrated highly elevated levels of scratching behavior compared with wild-type controls. A modest increase in the number of scratching bouts was also seen in mMCP6-deficient mice, while mMCP4-deficiency did not have any effect.

**Conclusion:**

Altogether, these findings identify a prominent role for the mast cell proteases, in particular CPA3, in the protection against itch induced by ET-1.

## Background

Itch is an unpleasant sensation that elicits the desire to scratch, and is most commonly caused either by light touch or by substances (pruritogens) that activate itch receptors on sensory neurons, either directly or indirectly. Histamine is the most extensively studied pruritogen, but a large and heterogeneous group of other pruritogens is also capable of inducing (histamine-independent) itch. Such histamine-independent itch can be challenging to treat effectively, especially in chronic pruritus [1]. Endothelin-1 (ET-1), a 21-amino acid peptide [2], is the most potent endogenous vasoconstrictor in the human cardiovascular system [3]. ET-1 can also induce pain when exogenously administered, and when lower doses are injected into the skin it can act as a powerful non-histaminergic pruritogen [4]. Two other ET isoforms have been identified, endothelin-2 (ET-2), and endothelin-3 (ET-3) [5], but ET-1 is the most widely expressed of these [6, 7]. ET-1 is mostly synthesized and released from vascular endothelial cells [2, 3] but is also produced by a variety of other cells, such as keratinocytes [8], vascular smooth muscle cells [9], dorsal root ganglia and spinal cord neurons [10, 11], macrophages, and mast cells [12, 13]. Endothelins bind to two subtypes of G protein-coupled receptors (GPCRs), the endothelin A receptor (ET_A_R) [14] and endothelin B receptor (ET_B_R) [15]. In vertebrates, the endothelin receptors are widely expressed throughout the body, and studies on both mice and humans have revealed that ET_A_R has the highest expression within the cardiovascular system and the lung, while ET_B_R is the predominant endothelin receptor in the brain ([16]; summarized in [3]). Due to its vasoconstriction properties and high expression within the cardiovascular system, ET-1 has an important role in regulating blood pressure [3] but ET-1 can also activate ET_A_Rs on primary afferents (ET_A_R/*Ednra* is expressed in 2-6% of primary afferents [17, 18]) and can transmit and potentiate both pain and itch [19–23]. Furthermore, ET_A_Rs are found on mast cells and ET-1 is capable of inducing mast cell degranulation [24, 25].

Mast cells are a part of the immune system and have an important role in host defense. When activated, they can degranulate and release a variety of different active mediators, many of which have pro-inflammatory or protective functions. Some of these substances, such as histamine, serotonin, leukotriene C4, and tryptase, can act directly on receptors on primary afferents as pruritogens [26, 27] and it has been suggested that mast cells are capable of producing ET-1 [12, 13]. Furthermore, mast cells can release proteases that affect ET-1 and its production, such as carboxypeptidase A3 (CPA3) that degrades ET-1 [28, 29] and chymase and Cathepsin E [30] that can convert the inactive precursor big-ET-1 into active ET-1 [31, 32]. It has previously been shown that mast cells are necessary for protection against otherwise fatal ET-1-induced toxicity in mice [33].

In the current study, we investigated the possible involvement of connective tissue mast cell proteases in ET-1-induced itch, induced by intradermal ET-1 administration at low (sub-lethal) doses. Our data reveal that CPA3 and, to a lesser extent, tryptase, play important roles in the protection against ET-1-induced scratching.

## Methods

### Generation of transgenic animals

Mice deficient in the mast cell proteases mMCP4 (*Mcpt4*^*-/-*^ [34]), mMCP6 (*Mcpt6*^*-/-*^ [35]) and CPA3 (*Cpa3*^*-/-*^ [36]) were generated as previously described. Mice deficient in all three proteases (*Mcpt4*^*-/-*^*Mcpt6*^*-/-*^*Cpa3*^*-/-*^) were then generated by intercrossing the above mentioned strains together on a C57BL/6 J genetic background [37]. The transgenic *Cpa3*^*Y356L,E378A*^ mouse strain, where the protease is rendered inactive by mutating the active sites of the enzyme, was generated as previously described [29]. The accuracy of the transgenic lines has been evaluated in previous analyses, where each mutation was shown to result in absence or inactivation of the respective protein [34–36, 38]. Mice were genotyped by PCR using the following primer combinations: mMCP-4 gene (*Mcpt4*) (forward primer, 59-CAA GGT CCA ACTAAC TCC CTT TGT GCT CC-39, wild-type [WT] reverse, 59- GGT GAT CTC CAG ATG GGC CAT GTA AGG GCG-39, knock-out [KO] reverse, 59-GGG CCA GCT CAT TCC TCC CAC TCA TGA TCT-39), mMCP-6 gene (*Tpsb2*) (forward primer, 59-TTT AGC TGG ACT CAG GCT GTG CTC CTC ACT-39, WT reverse, 59-CTC CTG AAT TGG AGC TAA CCC TGG GAT TCT-39, KO reverse, 59-GAC CAT GTG ATC GCG CTT CT-39), and CPA3 gene (*Cpa3*) (forward primer, 59-GGA CTG TTC ATC CCC AGG AAC C-39, reverse 1, 59-CTG GCG TGC TTT TCATTC TGG-39, reverse 2, 59-GTC CGG ACA CGC TGA ACT TG-39). Wild-type mice were on a C57BL/6 J genetic background.

### Behavior

All behavioral tests were performed on adult (> 7 weeks old) female and male mice. Control mice were gender- and age-matched wild-type mice (C57BL/6 J) housed in the same animal room. All behavior analyses were performed during the day (light) part of a 12 h day/night cycle, in a controlled environment of 20–24 °C and 45–65% humidity and by the same female investigator. All animal procedures were approved by the local ethical committee in Uppsala and followed the Directive 2010/63/EU of the European Parliament and of the Council, The Swedish Animal Welfare Act (SFS 1988:534), The Swedish Animal Welfare Ordinance (SFS 1988:539), and the provisions regarding the use of animals for scientific purposes: DFS 2004:15 and SJVFS 2012:26.

### Itch studies

50 μl of vehicle (0.9% saline, Fresenius Kabi) or endothelin-1 (20 pmol, Sigma-Aldrich) were injected intradermally in the back of the neck. Where the ET_A_R antagonist BQ-123 was used, 10 pmol of endothelin-1 were injected, with or without 10 nmol of BQ-123 (Sigma-Aldrich) in one intradermal injection. Each mouse was then placed in a cage with wooden chips and recorded for 1 h using a digital video camera. Afterwards, AniTracker® v1.2 was used to score the videos for the duration and frequency of grooming and scratching behavior. A scratching episode was defined as a bout of scratching by either hind paw; from the time point it was lifted until placed back on the ground. The videos were scored by observers blinded to the genotype and treatment given. The results for each group are expressed as the mean frequency of scratching episodes/60 min, the mean duration of scratching episodes/60 min, and, where relevant, as the mean length of scratching episodes (total duration/total frequency) and as the mean frequency of scratching episodes per each 10 min interval. Results are presented as mean ± SEM.

### Statistics

For all sets of data, Gaussian distribution was assessed by a Shapiro-Wilks test. Student’s *t* test, two-tailed, was used to compare two groups. When the groups were more than two, 1-way ANOVA and either Dunnett’s or Bonferroni multiple comparison post hoc tests were used for parametric data, for non-parametric data Kruskal-Wallis and Dunn’s multiple comparison post hoc test were used. Two-way repeated measurement ANOVA with Bonferroni multiple comparison post hoc test was used to analyze the development of scratching frequency over time. Grubbs’ test was used to identify significant outliers (https://www.graphpad.com/quickcalcs/Grubbs1.cfm, GraphPad Software, Inc.). All other calculations were performed using Prism version 5.04 (GraphPad Software, Inc., San Diego, CA). Results are presented as mean ± S.E.M. *P* values of < 0.05 were considered significant. In the figures, * refers to *P* ≤ 0.05, ** refers to *P* ≤ 0.01, *** refers to *P* ≤ 0.001, **** refers to *P* ≤ 0.0001 and n.s. stands for not significant. Lower case “*n*” indicates the number of animals used in a set of experiments.

## Results

### Mcpt6^-/-^ mice scratch more frequently than controls in response to vehicle injection

First, *Mcpt4*^*-/-*^, *Mcpt6*^*-/-*^, *Cpa3*^*Y356L,E378A*^, *Mcpt4*^*-/-*^*Mcpt6*^*-/-*^*Cpa3*^*-/-*^ mice and C57BL/6 J controls were injected with vehicle (0.9% sterile saline) to evaluate the basal scratching behavior resulting from handling and injection (Supplementary Fig. 1). The protease-deficient mice did not differ from controls in response to saline, with the exception of *Mcpt6*^*-/-*^ mice that scratched slightly more frequently than controls (Supplementary Fig. 1a, *P* < 0.05), but no differences were observed with regard to scratching duration in 60 min or mean length of scratching episodes (Supplementary Fig. 1b-1c). This indicates that *Mcpt6*^*-/-*^ mice may in general be more sensitive to intradermal injections, irrespective of substance injected.

### Blocking the ET_A_ receptor significantly decreases ET-1-induced scratching

**Fig. 1:**
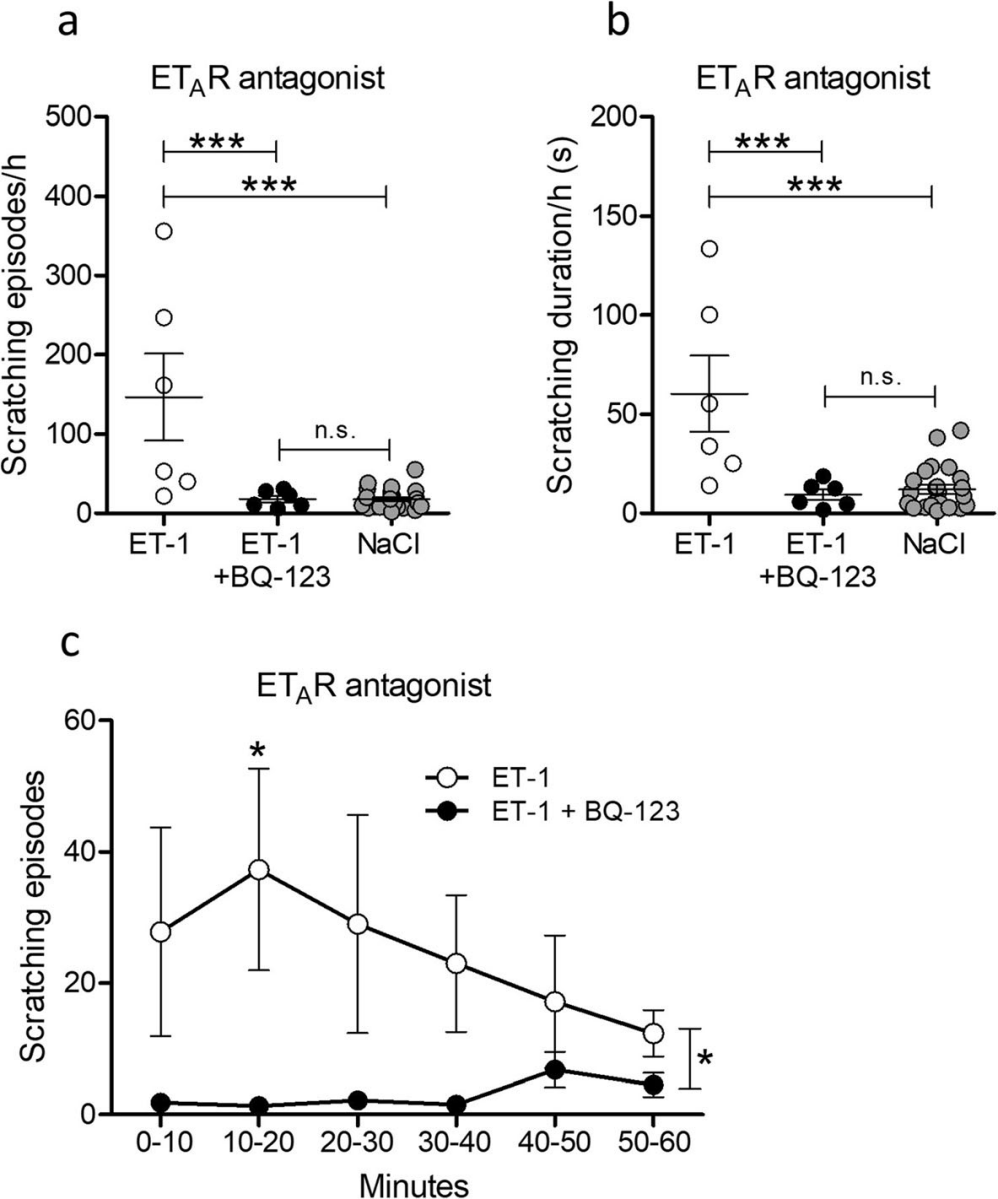
ET-1-induced scratching is effectively abolished by antagonizing the ET_A_ receptor. Wild-type (C57BL/6 J) mice were injected intradermally in the nape with either ET-1 (10 pmol, *n* = 6) or ET-1 together with ET_A_R antagonist BQ-123 (10 pmol + 10 nmol, *n* = 6). **a**, **b** BQ-123 was very effective in reducing the total scratching frequency and duration and the scratching behavior was similar to vehicle-injected (saline) animals (*n* = 24, data from Supplementary Figure 1). **c** When the frequency results from (**a**) were analyzed over time with the BQ-123 treatment as a factor, it was seen that antagonist-treated mice scratched significantly less overall (indicated by significance bar on the far right), and post hoc analysis showed significant difference between treatment groups in the interval between 10 and 20 min. Results are presented as mean ± SEM. *P ≤ 0.05; ***P ≤ 0.001; n.s., not significant; 1-way ANOVA with Bonferroni multiple comparison (**a**, **b**); 2-way ANOVA with Bonferroni multiple comparison (**c**). Vehicle data in (**a**, **b**) is the same data as presented in Supplementary Figure 1a-1b

ET-1 transmits itch by binding to and activating ET_A_Rs on primary afferents. To exclude possible off-target effects by ET-1, we administrated the peptide in wild-type mice with or without the selective ET_A_R antagonist BQ-123 [39] and evaluated the scratching behavior. Various different dosage combinations of ET-1 and BQ-123 have been reported, and we decided to follow the example of Trentin et al. [20], where 10 pmol of ET-1 were injected together with 10 nmol of BQ-123. The antagonist was effective in reducing the total scratching, with regards to both the frequency of scratching bouts (Fig. 1a, *P* < 0.001) and scratching duration (Fig. 1b, *P* < 0.001). For reference, the scratching levels of both treatment groups were compared with vehicle-injected wild-type controls (data from Supplementary Fig. 1a-1b). No statistical difference was found between antagonist-treated and vehicle-injected animals (Fig. 1a, b; *P* > 0.05) but a significant difference between ET-1-treated and vehicle-injected mice was seen (Fig. 1a, b; *P* < 0.001). Analysis of scratching frequency over time and in 10-min intervals with the antagonist treatment as a factor revealed that scratching was almost completely abolished within the first 40 min in BQ-123-treated mice (Fig. 1c). Post hoc analysis showed that in the time interval between 10 and 20 min, the difference in scratching frequency between treatment groups became significant (Fig. 1c, *P* < 0.05). Overall analysis on scratching frequency with time showed that BQ-123 was effective in reducing scratching (Fig. 1c, *P* < 0.05). Taken together, the above results show that BQ-123 blocks ET-1-induced scratching behavior and that ET-1-induced scratching depends specifically on ET_A_R activation. In the group that only received ET-1 (10 pmol), half of the animals showed only a modest increase in scratching behavior (Fig. 1a, b) and therefore it was decided to increase the dose to 20 pmol in subsequent experiments.

### Mcpt4^-/-^Mcpt6^-/-^Cpa3^-/-^ mice show profoundly enhanced scratching behavior when provoked with ET-1

**Fig. 2:**
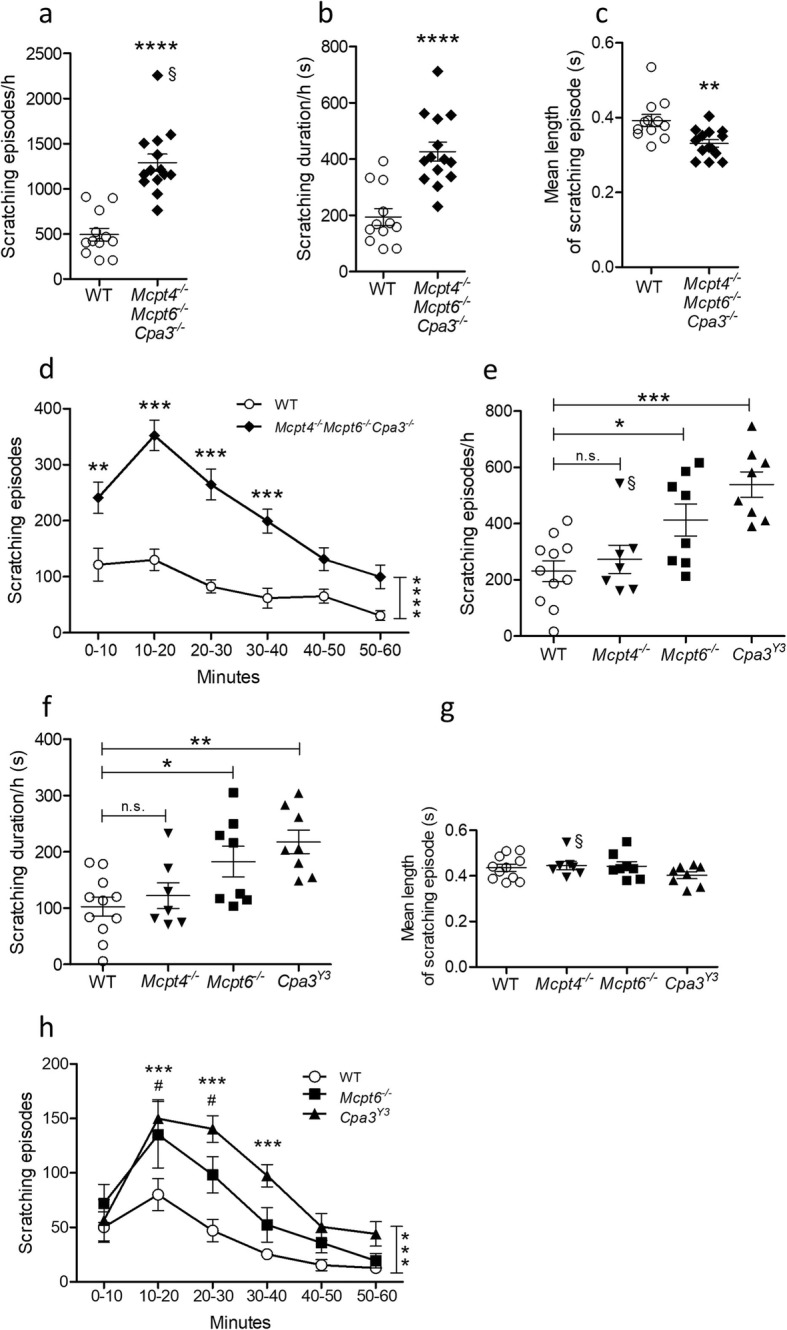
The mast cell protease-deficient mouse lines Mcpt4^-/-^Mcpt6^-/-^Cpa3^-/-^, Cpa3^Y356L,E378A^, and Mcpt6^-/-^ scratch more than wild-type controls when injected with ET-1. **a** When injected with ET-1 (20 pmol), *Mcpt4*^*-/-*^*Mcpt6*^*-/-*^*Cpa3*^*-/-*^ mice (*n* = 14) had a much higher number of scratching episodes in 60 min than wild-type controls (WT, *n* = 12). **b ***Mcpt4*^*-/-*^*Mcpt6*^*-/-*^*Cpa3*^*-/-*^ mice spent more total time scratching during the 60 min test. **c** The *Mcpt4*^*-/-*^*Mcpt6*^*-/-*^*Cpa3*^*-/-*^ mice also had shorter scratching episodes on average than controls. **d** The results from (**a**) analyzed over time. The *Mcpt4*^*-/-*^*Mcpt6*^*-/-*^*Cpa3*^*-/-*^ mice scratched more frequently than controls overall (vertical significance bar), and post hoc analysis showed that they scratched significantly more in the first four intervals of the test. **e** Both *Mcpt6*^*-/-*^ (*n* = 8) and *Cpa3*^*Y356L,E378A*^ mice (*Cpa3*^*Y3*^, *n* = 8) scratched more frequently in 60 min than wild-type controls (*n* = 11) when injected with ET-1. *Mcpt4*^*-/-*^ mice (*n* = 7) did not differ from controls in their scratching behavior. **f***Mcpt6*^*-/-*^ and *Cpa3*^*Y356L,E378A*^ mice also spent more time scratching after ET-1 injection than controls. **g** But no difference was seen in the mean length of scratching episodes. **h** The results from (**e**) analyzed over time. Both *Mcpt6*^*-/-*^ and *Cpa3*^*Y356L,E378A*^ mice scratched more than controls over the duration of the test (indicated by vertical significance bar). Further analysis showed that *Cpa3*^*Y356L,E378A*^ mice scratched more than controls in the intervals between 10 and 40 min (indicated by stars), and *Mcpt6*^*-/-*^ mice scratched significantly more between 10 and 30 min (indicated by #). Results are presented as mean ± SEM. #/*P ≤ 0.05; **P ≤ 0.01; ***P ≤ 0.001; ****P ≤ 0.0001; n.s, not significant; Student’s *t* test (**a-c**); 2-way ANOVA with Bonferroni multiple comparison (**d**, **h**); 1-way ANOVA (**e-g**). Statistical outliers as identified by Grubb’s test are indicated with the symbol §



Mouse connective tissue mast cells express mMCP4, mMCP5, mMCP6, mMCP7, and CPA3 [40] and to investigate how the loss of connective tissue mast cell-specific proteases affects ET-1-induced scratch behavior, we tested the *Mcpt4*^*-/-*^*Mcpt6*^*-/-*^*Cpa3*^*-/-*^ mouse line by injecting ET-1 intradermally in the nape and compared them with wild-type controls (Fig. 2a, d). The *Mcpt4*^*-/-*^*Mcpt6*^*-/-*^*Cpa3*^*-/-*^ line lacks the protease mMCP5 as the protein depends on CPA3 for storage [36], and mMCP7 is only expressed in low levels in mature mast cells [37], rendering a line that is almost devoid of connective tissue mast cell-specific proteases. In the *Mcpt4*^*-/-*^*Mcpt6*^*-/-*^*Cpa3*^*-/-*^ mice, a large increase in scratching was seen; in 1 h the protease-deficient mice scratched on average 1214.0 ± 66 times while the controls had 492.0 ± 70 scratching episodes during the same time period (Fig. 2a, *P* < 0.0001). The *Mcpt4*^*-/-*^*Mcpt6*^*-/-*^*Cpa3*^*-/-*^ animal displaying the most exaggerated scratching behavior had more than 2200 scratching episodes during the 60 min; this animal was found to be a significant outlier according to Grubb’s test. However, including or excluding that animal did not affect the statistical significance of the result (*P* < 0.0001 in both instances, the animal is indicated with a § in the respective figure). When total scratching duration was analyzed (Fig. 2b), the results were similar to those seen for scratching frequency; the protease-deficient mice spent significantly more time scratching than controls (*P* < 0.0001). The *Mcpt4*^*-/-*^*Mcpt6*^*-/-*^*Cpa3*^*-/-*^ animals also had shorter scratching episodes on average (Fig. 2c, *P* = 0.0025). When the scratching episode frequency was analyzed over time and in 10-min intervals (Fig. 2d), it was seen that the *Mcpt4*^*-/-*^*Mcpt6*^*-/-*^*Cpa3*^*-/-*^ knockouts scratched more over time than the controls overall (*P* < 0.0001) and post hoc testing revealed that the difference was statistically significant during the first 40 min of the test (0-10 min: *P* < 0.01, 10-40 min: *P* < 0.001). The above results indicate that the mast cell-specific proteases play an important protective role in ET-1-induced scratch behavior. A video demonstrating the typical scratching behavior 10 min. after ET-1 injection in a wild-type control and a *Mcpt4*^*-/-*^*Mcpt6*^*-/-*^*Cpa3*^*-/-*^ mouse can be found in online Supplementary Materials (Movie S1).

**Additional file 2: Movie S1.**



### The Mcpt6^-/-^ and Cpa3^Y356L,E378A^ mouse lines have an exaggerated scratching response to ET-1

Next, we aimed to identify which of the mast cell proteases was/were responsible for the protective effect against ET-1-induced scratching. We therefore evaluated the pruritic effects of an intradermal injection of ET-1 on the single mast cell protease knock-out lines *Mcpt4*^*-/-*^ and *Mcpt6*^*-/-*^, as well as the transgenic line with enzymatically inactive CPA3 (*Cpa3*^*Y356L,E378A*^) (Fig. 2e, h).

*Cpa3*^*Y356L,E378A*^ mice were initially used in this study instead of *Cpa3*^*-/-*^ mice, since it has been shown that *Cpa3*^*-/-*^ mice also lack mouse mast cell protease 5 (mMCP5) [36] and are thus functionally double-deficient in enzyme activity. CPA3 has been shown to protect against ET-1 toxicity in vivo [29]. Here, we noted that *Cpa3*^*Y356L,E378A*^ mice scratched more frequently than controls during the 60 min test (Fig. 2e, *P* < 0.001). When scratching duration was analyzed, the findings were similar (Fig. 2f); the *Cpa3*^*Y356L,E378A*^ animals spent more total time scratching than controls (*P* < 0.01). No difference was observed between groups in the mean length of scratching episodes (Fig. 2g, *P* > 0.05).

mMCP4 is a chymase, and it has been demonstrated that mast cell chymase can cleave the inflammatory neuropeptides substance P and vasoactive intestinal peptide (VIP [41, 42]), both of which can induce itch and degranulate mast cells [43–45]. Despite these potential abilities of mMCP4 to protect against itch, we saw no difference in scratching frequency, scratching duration or mean episode length in *Mcpt4*^*-/-*^ mice compared with controls (Fig. 2e, g; *P* > 0.05), indicating that mMCP4 is not involved in regulating ET-1-induced scratching. One *Mcpt4*^*-/-*^ animal was a significant outlier in scratching frequency (Fig. 2e) and another was an outlier in the mean length of scratching episodes (Fig. 2g). However, including or excluding these individuals did not have any effects on the results of the statistical comparison with controls, i.e., no differences were observed (*P* > 0.05, the animals are indicated with a § in the respective figures).

Finally, we investigated the role of the tryptase mMCP6 in ET-1-induced scratching. The analysis showed that *Mcpt6*^*-/-*^ mice scratched more frequently and spent more time scratching than controls (Fig. 2e, f; *P* < 0.05), but the statistical significance was lower than seen between *Cpa3*^*Y356L,E378A*^ mice and controls.

The development of the scratching behavior for all single protease transgenic lines was also analyzed over time in 10-min intervals (Fig. 2 h). The analysis revealed that both *Cpa3*^*Y356L,E378A*^ and *Mcpt6*^*-/-*^ animals had more frequent scratching episodes than controls overall during the test (*P* = 0.0002), but no difference was observed in *Mcpt4*^*-/-*^ animals (data not shown). Post hoc testing on the individual intervals showed that the scratching episode frequency of *Cpa3*^*Y356L,E378A*^ mice was elevated compared with controls in the intervals between 10 and 40 min (Fig. 2h, *P* < 0.001). *Mcpt6*^*-/-*^ mice had elevated scratching frequency compared with controls in the intervals between 10-30 min (Fig. 2h, *P* < 0.05).

Taken together, the above data indicate that the mast cell proteases CPA3, and to a smaller extent, mMCP6, play a role in attenuating ET-1-induced scratching, while mMCP4 does not.

### mMCP5 deficiency does not have an effect on ET-1-induced scratch behavior

**Fig. 3:**
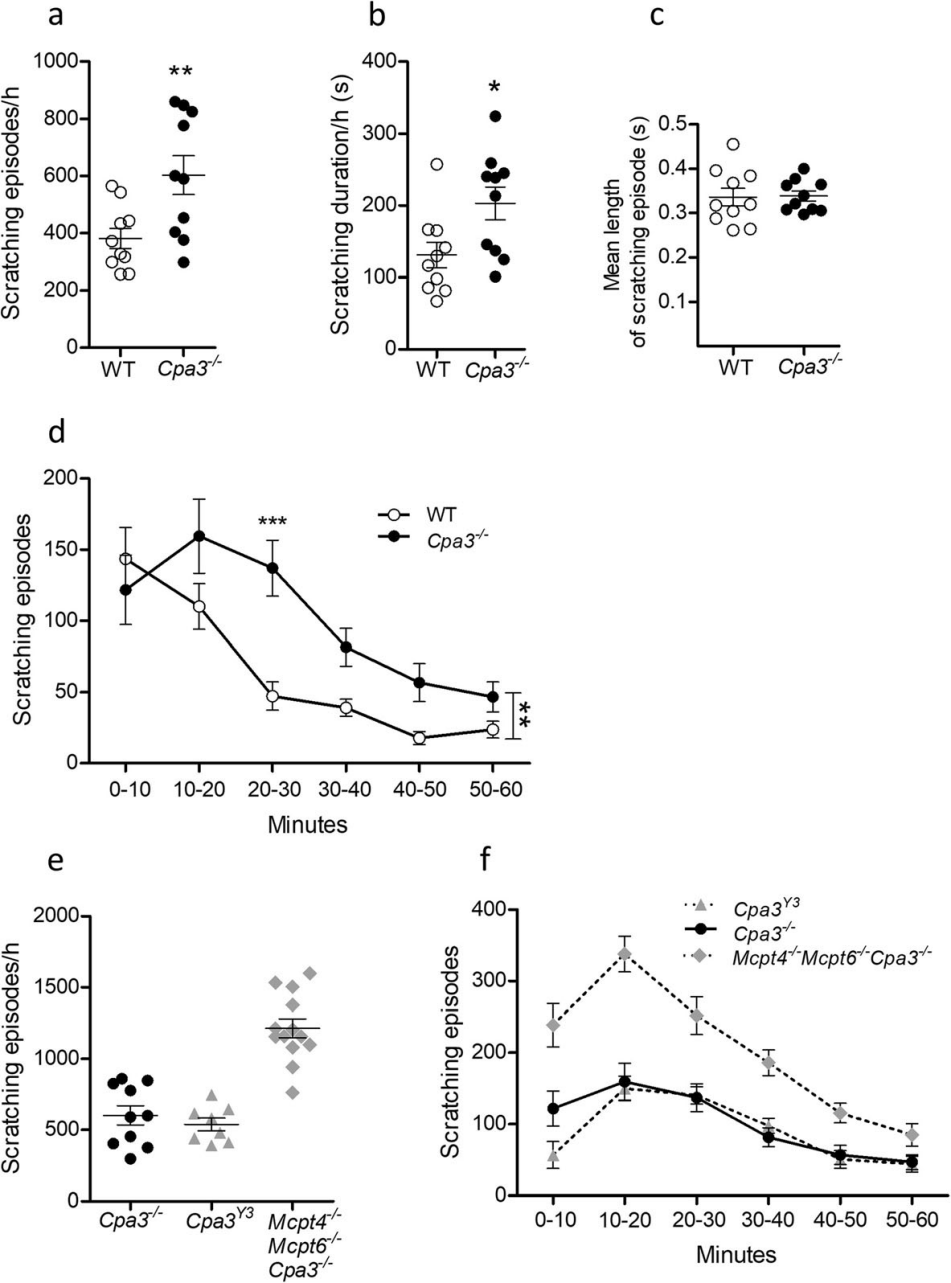
ET-1-induced scratching is not affected by mMCP5 deficiency. **a***Cpa3*^*-/-*^ mice (*n* = 10) are deficient in both CPA3 and mMCP5, and had more frequent scratching bouts in 60 min than wild-type controls (WT, *n* = 10) when injected with ET-1 (20 pmol). **b***Cpa3*^*-/-*^ mice also spent more time scratching during the 60 min. **c** No difference was seen between genotypes in the mean length of scratching episodes. **d** When the frequency results from (**a**) were analyzed over time, it was seen that *Cpa3*^*-/-*^ mice scratched significantly more than controls overall (indicated by vertical significance bar), and post hoc analysis showed a significant difference in the interval between 20 and 30 min. **e**, **f** Visual comparison of the total frequency of scratching episodes and scratching frequency development over time, between *Cpa3*^*-/-*^ mice (data from (a)), *Cpa3*^*Y356L,E378A*^ mice (*Cpa3*^*Y3*^, data from Fig. 2e) and *Mcpt4*^*-/-*^*Mcpt6*^*-/-*^*Cpa3*^*-/-*^ mice (data from Fig. 2a). The two CPA3-deficient mouse lines follow a similar scratching pattern and magnitude, while the *Mcpt4*^*-/-*^*Mcpt6*^*-/-*^*Cpa3*^*-/-*^ mice demonstrate more exaggerated behavior. Results are presented as mean ± SEM. *P ≤ 0.05, **P ≤ 0.01, ***P ≤ 0.001, Student’s *t* test (**a-c**), 2-way ANOVA with Bonferroni multiple comparison (**d**)

It has previously been shown that CPA3 catalyzes the breakdown of ET-1 in vitro [29] and is important in reducing the toxic effects of ET-1 administered intraperitoneally [29, 33], and our results indicate that CPA3 is also involved in attenuating the pruritic effects of ET-1 administered intradermally. The question still remained if the extensive scratching behavior demonstrated by the *Mcpt4*^*-/-*^*Mcpt6*^*-/-*^*Cpa3*^*-/-*^ mouse line was exclusively the result of lacking CPA3 and mMCP6. As mentioned above, when CPA3 is removed from the mast cell, mMCP5 (elastase) is lost as well (at the protein but not mRNA level) [36]. The *Cpa3*^*Y356L,E378A*^ mouse line expresses CPA3 that is devoid of enzymatic activity and possesses functional mMCP5, while the *Mcpt4*^*-/-*^*Mcpt6*^*-/-*^*Cpa3*^*-/-*^ mouse line has neither CPA3 nor mMCP5. This prompted the question if lacking mMCP5 had any effect on ET-1-induced scratching. To test this possibility, we performed the ET-1 test on the *Cpa3*^*-/-*^ mouse line, which lacks both CPA3 and mMCP5 (Fig. 3a, d). The *Cpa3*^*-/-*^ mice had a higher frequency of scratching bouts than their wild-type counterparts (Fig. 3a, *P* = 0.0094), and their scratching duration was elevated vs. controls (Fig. 3b, *P* = 0.022). There was no difference between *Cpa3*^*-/-*^ mice and controls in the mean length of scratching episodes (Fig. 3c, *P* = 0.904). When the scratching frequency was analyzed over time and in 10-min intervals (Fig. 3d), it was seen that the *Cpa3*^*-/-*^ mice scratched more than controls overall (*P* = 0.0094). Post hoc analysis on the intervals revealed that *Cpa3*^*-/-*^ mice displayed more scratching episodes than controls in the interval between 20 and 30 min (*P* < 0.001).

Direct statistical comparison between groups tested at different time points, using different batches of ET-1, is not feasible since ET-1 is a very potent peptide that degrades with time despite freezer storage. This was confirmed by comparing the wild-type controls that were used in three sets of experiments using different batches of ET-1, and small but significant differences were found between the groups (data not shown). However, a visual comparison of the total scratching frequency in *Cpa3*^*-/-*^ mice, *Cpa3*^*Y356L,E378A*^ mice and *Mcpt4*^*-/-*^*Mcpt6*^*-/-*^*Cpa3*^*-/-*^ mice (Fig. 3e), and how the scratching frequency develops over time (Fig. 3f), suggests that *Cpa3*^*Y356L,E378A*^ and *Cpa3*^*-/-*^ animals have a similar pattern of scratching behavior and magnitude, while the *Mcpt4*^*-/-*^*Mcpt6*^*-/-*^*Cpa3*^*-/-*^ mice show more exaggerated behavior. Together, these findings indicate that mMCP5 does not contribute to the protection against the scratching behavior seen in the *Mcpt4*^*-/-*^*Mcpt6*^*-/-*^*Cpa3*^*-/-*^ mice.

## Discussion

Here, we investigated the possible involvement of mouse connective tissue mast cell-specific proteases in the scratching behavior induced by ET-1. Both mMCP6 and CPA3 deficiency, as well as the combined loss of mMCP4, mMCP6 and CPA3, resulted in significantly increased scratching behavior compared with wild-type controls, while mMCP4-deficiency alone did not have an effect. We could also conclude that mMCP5 is not involved in ET-1-induced scratching behavior.

### ET-1 induces itch and pain through the ET_A_ receptor on primary afferents

ET-1 binds to and activates the G protein-coupled receptors ET_A_R and ET_B_R [46], and in humans it can cause pain, hyperalgesia, and intense pruritus when exogenously administered, depending on the dose and the route of administration [22, 23, 47]. In the periphery, it has been reported that ET_A_R is mainly responsible for pain and itch transmission, while ET_B_R has generally been coupled to analgesic and antipruritic effects [20, 48, 49]. Similar to what has been seen with other algogens, central administration of ET-1 induces analgesia, which is mediated through both ET_A_R and ET_B_R [50]. Since animals cannot describe their sensations, a useful behavioral model to distinguish between algogens and pruritogens has been developed in mice: the mouse cheek model [51]. In this model, a substance is injected intradermally in the cheek and if the substance is an algogen the mouse responds to the pain by wiping the face with its forepaws, while pruritogens will elicit scratches by the hind paws. When ET-1 was tested in the cheek test, the mice exhibited both pain and itch behaviors [21], indicating that ET-1 is a substance that can induce both sensations. This suggests that the particular sensation experienced upon injection of ET-1 has certain painful properties experienced by the animals, a feeling that human volunteers have described as “burning itch” [22]. Furthermore, ET-1 is capable of degranulating mast cells through ET_A_R, causing release of pruritogens such as histamine and leukotriene C4 [24, 25, 52]. However, ET-1-induced itch is generally considered to be non-histaminergic. It depends upon other neuronal signaling mechanisms than histamine [49, 53] and histamine 1 receptor antagonists have little effect on the scratching behavior [20, 49]. Degranulation also releases large amounts of mast cell proteases, which have been reported to have both protective and inflammatory properties [40, 54, 55].

### Multiple mast cell protease deficiency and ET-1-induced scratching

*Mcpt4*^*-/-*^*Mcpt6*^*-/-*^*Cpa3*^*-/-*^ mice lack all connective tissue type mast cell-specific proteases, and this results in abnormal mast cell secretory granule composition and morphology, as well as reduction in heparin content [37]. It is therefore not surprising that these animals have abnormal physiological, and consequently, behavioral, responses to provocation. In our study, we saw greatly increased scratching behavior upon ET-1 injection in *Mcpt4*^*-/-*^*Mcpt6*^*-/-*^*Cpa3*^*-/-*^ mice compared with controls, indicating that mast cell-specific proteases serve an important function in attenuating the itch induced by the peptide. In order to evaluate the contribution from the individual proteases, however, we also assessed mice with single deficiency of the respective proteases.

These analyses revealed that both *Cpa3*^*Y356L,E378A*^ and *Mcpt6*^*-/-*^ animals scratched significantly more than controls in response to ET-1, but the *Mcpt4*^*-/-*^*Mcpt6*^*-/-*^*Cpa3*^*-/-*^ mouse line exhibited scratching that was of an even greater magnitude. Since the *Mcpt4*^*-/-*^*Mcpt6*^*-/-*^*Cpa3*^*-/-*^ mouse line is lacking both mMCP6 and CPA3, a certain additive effect was not unexpected but this prompted the question if the intense scratching behavior was only due to the lack of these two proteases, or if mMCP5-deficiency also played a role. mMCP5 is a mast cell elastase which has not been extensively studied and no connection to itch has been reported, but it has been shown to have a role in mediating epidermal burn injuries in mice [56]. Since *Mcpt4*^*-/-*^*Mcpt6*^*-/-*^*Cpa3*^*-/-*^ mice lack CPA3, they also lose the ability to store the elastase mMCP5. To investigate the potential properties of mMCP5, the ET-1 test was also performed on *Cpa3*^*-/-*^ mice (that lack both CPA3 and mMCP5), to see if their scratching behavior would follow a similar pattern and magnitude as seen in *Cpa3*^*Y356L,E378A*^ mice (that possess mMCP5 but enzymatically non-functional CPA3) or *Mcpt4*^*-/-*^*Mcpt6*^*-/-*^*Cpa3*^*-/-*^ mice. However, the results from the *Cpa3*^*-/-*^ mice did not resemble the greatly enhanced scratching seen in the multiple protease-deficient mice. This indicates that mMCP5 does not have an important role in the protection against ET-1-induced scratching.

### CPA3 and ET-1-induced scratch behavior

CPA3 is not as well studied as mMCP4 and mMCP6, and has mainly received attention for its role in host defense by cleaving a particular type of toxins, sarafotoxins, that can be found in snake and bee venom [29, 57]. Sarafotoxins belong to the endothelin family and it has been demonstrated that CPA3 cleaves both sarafotoxin S6b and ET-1 at the C-terminal, which results in inactive and non-toxic products [29]. Mast cell chymase also cleaves those substrates, but at an internal location, which is not sufficient to reduce the toxicity [29]. CPA3 is necessary to protect against otherwise fatal ET-1 toxicity when it is injected intraperitoneally in doses between 1 and 3 nmols [29, 33]. In the current study, a considerably lower dose of ET-1 (20 pmol) and another route of administration (intradermal) were used to investigate ET-1-induced itch instead of toxicity, and our results show that CPA3-deficiency results in significantly exaggerated scratching behavior compared with controls. This result was seen in all the mouse lines that lack functional CPA3: *Cpa3*^*Y356L,E378A*^, *Cpa3*^*-/-*^, and *Mcpt4*^*-/-*^*Mcpt6*^*-/-*^*Cpa3*^*-/-*^. This suggests that CPA3 has an important role in protection against exogenously administered ET-1 with regard to pruritus.

### mMCP4 and ET-1-induced scratch behavior

Chymase has the capability to produce ET-1 by cleaving the precursor peptide Big-ET-1 in vivo [58]. Chymase has also been reported to cleave known itch mediators such as interleukins 6, 13, and 33 (IL-6, IL-13, IL-33 [59, 60]) as well as substance P and VIP [41–43]. Despite these properties, there is little data that links mMCP4 directly to pruritus, but studies have shown that chymase inhibition is successful in attenuating itch and skin inflammation in a mouse AD model [61, 62]. In our study, no effects of chymase on ET-1-induced scratching behavior were observed. This is in accordance with a previous study showing that mMCP4 knockouts have the same resistance to ET-1-like toxicity (induced by sarafotoxins) as wild-type controls, while the toxicity was fatal in mast cell-deficient mice [57].

### mMCP6 and ET-1-induced scratch behavior

*Mcpt6*^*-/-*^ mice demonstrated higher levels of scratching behavior than controls in response to ET-1. This could potentially be explained by direct ET-1 inactivation catalyzed by mMCP6. Alternatively, mMCP6 could affect signaling events downstream of the binding of ET-1 to its receptor. Human tryptase has been reported to cleave CGRP in vitro in an efficient manner [63] and ET-1 induces the release of CGRP and glutamate from primary afferents through ET_A_R activation [64]. Since CGRP has been implicated in itch modulation [65, 66], mMCP6 may have an effect on ET-1-induced scratch behavior indirectly, through the degradation of CGRP. However, the *Mcpt6*^*-/-*^ mice may also be more sensitive to the injection of an inert vehicle (Supplementary Fig. 1a), which further complicates the interpretation of their scratching behavior.

## Conclusions

Here, we have investigated the effects of connective tissue mast cell protease deficiency on ET-1-induced scratching behavior. We found that the mast cell carboxypeptidase CPA3 was critical for attenuating ET-1-induced scratching, and our data also indicate that mast cell tryptase can contribute to such protection. Importantly, the protease expression profile of mouse and human skin mast cells is highly analogous and the substrate specificities of the corresponding mouse and human mast cell proteases are closely related [40, 55], and it is therefore likely that human mast cell proteases have a similar impact on itch as seen in the approach adopted here. Altogether, these findings establish a novel principle for how itch responses can be regulated. Intriguingly, since mast cells are also strongly implicated in promoting itch signaling (e.g., by secreting histamine and serotonin), our present investigations introduce the notion that mast cells can both promote and attenuate itch.

## Supplementary information

**Additional file 1: Figure S1.***mMCP6-deficient mice potentially scratch more than controls in response to vehicle injection.***a)***Mcpt6*^*-/-*^ mice (n = 6) had slightly more frequent scratching bouts in 60 minutes than wild-type controls (WT, n = 24) when injected intradermally with vehicle (0.9% saline, 50 μL), while *Mcpt4*^*-/-*^ (n = 9), *Cpa3*^*Y356L,E378A*^ (*Cpa3*^*Y3*^, n = 8) and *Mcpt4*^*-/-*^*Mcpt6*^*-/-*^*Cpa3*^*-/-*^ mice (n = 10) did not. **b)** No difference was seen between groups in scratching duration after vehicle injection, **c)** or in mean length of scratching episodes. Vehicle data for controls are pooled from three different experiments. Results are presented as mean ± SEM. *P ≤ 0.05, Kruskal-Wallis. Statistical outliers as identified by Grubb’s test are indicated with the symbol §.

## Data Availability

All data generated or analyzed during this study are included in this published article (and its supplementary information files) and available from the corresponding author on reasonable request.
