# Supplementary material for: Mouse connective tissue mast cell proteases tryptase and carboxypeptidase A3 play protective roles in itch induced by endothelin-1
Source: J Neuroinflammation. 2020 Apr 22;17:123. doi: 10.1186/s12974-020-01795-4 (PMC7175568; doi:10.1186/s12974-020-01795-4)
Supplement: Supplementary file 2 — Additional file 1: Figure S1.mMCP6-deficient mice potentially scratch more than controls in response to vehicle injection.a)Mcpt6-/- mice (n = 6) had slightly more frequent scratching bouts in 60 minutes than wild-type controls (WT, n = 24) when injected intradermally with vehicle (0.9% saline, 50 μL), while Mcpt4-/- (n = 9), Cpa3Y356L,E378A (Cpa3Y3, n = 8) and Mcpt4-/-Mcpt6-/-Cpa3-/- mice (n = 10) did not. b) No difference was seen between groups in scratching duration after vehicle injection, c) or in mean length of scratching episodes. Vehicle data for controls are pooled from three different experiments. Results are presented as mean ± SEM. *P ≤ 0.05, Kruskal-Wallis. Statistical outliers as identified by Grubb’s test are indicated with the symbol §. [file 12974_2020_1795_MOESM1_ESM.docx]

Supplementary material


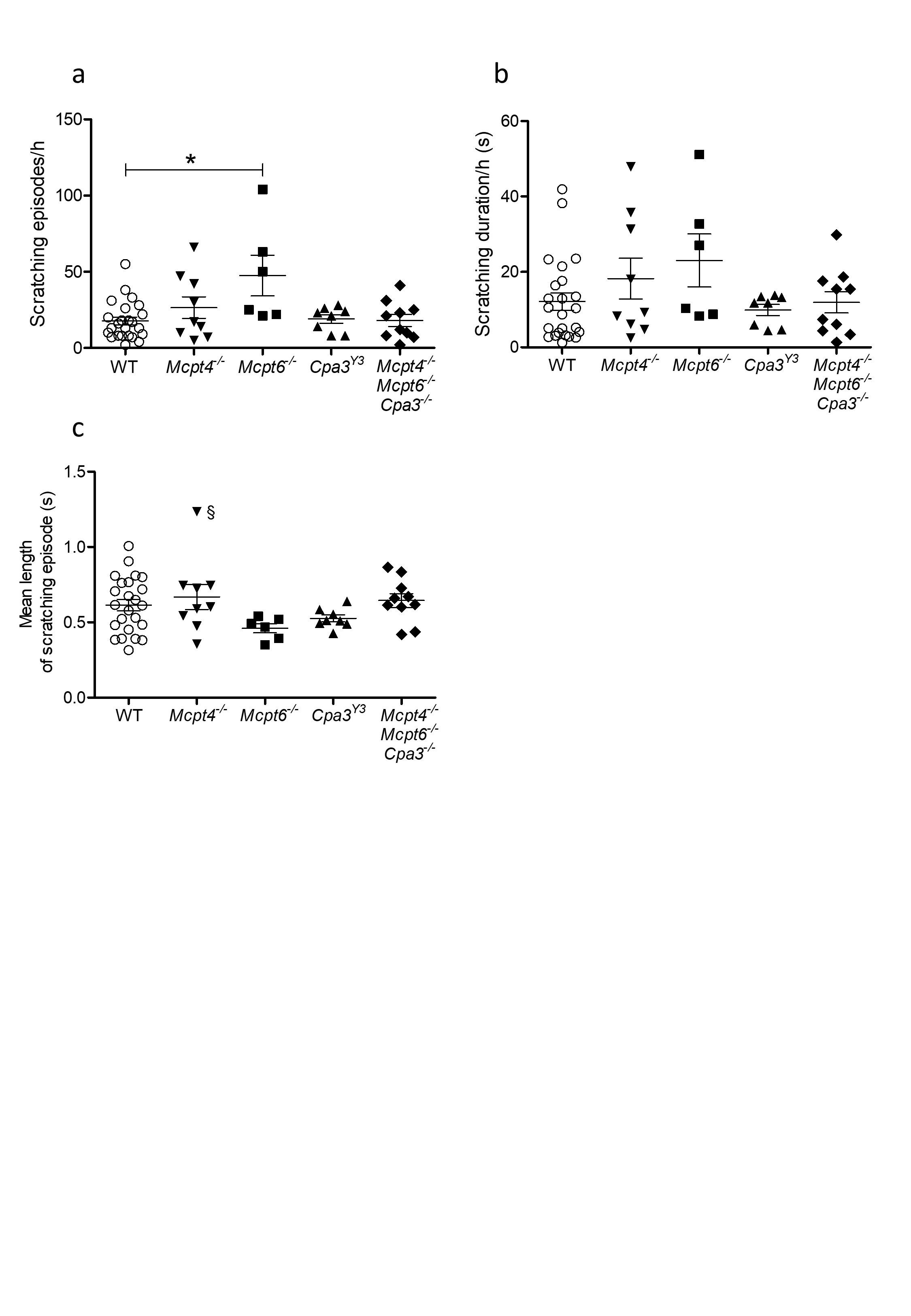


**Supplementary Figure S1.** *mMCP6-deficient mice potentially scratch more than controls in response to vehicle injection.* **a)** *Mcpt6^-/-^* mice (n = 6) had slightly more frequent scratching bouts in 60 minutes than wild-type controls (WT, n = 24) when injected intradermally with vehicle (0.9% saline, 50 µL), while *Mcpt4^-/-^* (n = 9), *Cpa3^Y356L,E378A^* (*Cpa3^Y3^*, n = 8) and *Mcpt4^-/-^Mcpt6^-/-^Cpa3^-/-^* mice (n = 10) did not. **b)** No difference was seen between groups in scratching duration after vehicle injection, **c)** or in mean length of scratching episodes. Vehicle data for controls are pooled from three different experiments. Results are presented as mean ± SEM. *P ≤ 0.05, Kruskal-Wallis. Statistical outliers as identified by Grubb’s test are indicated with the symbol §.
